# Facile Surface Depolymerization Promotes the Welding of Hard Epoxy Vitrimer

**DOI:** 10.3390/ma15134488

**Published:** 2022-06-25

**Authors:** Le An, Wenzhe Zhao

**Affiliations:** State Key Laboratory for Strength and Vibration of Mechanical Structures, Department of Engineering Mechanics, Xi’an Jiaotong University, Xi’an 710049, China; wenzhe0301@stu.xjtu.edu.cn

**Keywords:** epoxy vitrimer, surface depolymerization, welding, bond exchange reactions

## Abstract

Welding via bond exchange reactions has provided advances in obtaining high-quality joining performance. However, the reported welding method requires a relatively high press force, and challenges are still encountered in welding hard vitrimer. In this work, a facile surface depolymerization strategy was introduced to weld high-performance epoxy vitrimer. The vitrimers were firstly dissolved into ethylene glycol for depolymerization based on the solvent-assisted bond exchange reactions. Then, the depolymerized vitrimers were welded under heat and press force. The effect of the depolymerizing time, welding pressure, welding temperature and welding time on the welding strength were further investigated. It was found that there were optimal values for the depolymerizing time, welding pressure, and welding temperature, respectively, for the welding strength, while the welding strength increased with increasing welding time. Through facile surface degradation, the welding pressure was highly reduced, while the welding strength was increased. With surface depolymerization, the welding strength was 1.55-times higher, but the magnitude of press force was 1/1000-times than that with no surface depolymerization. It is elucidative that surface depolymerization can be used to weld hard vitrimer composites alongside reducing the press force effectively.

## 1. Introduction

Vitrimer is a novel kind of thermoset, where dynamic covalent bonds introduced into the cross-linked network can break, re-connect or exchange under external stimuli [1,2,3,4,5]. This type of polymer not only behaves as a conventional thermoset in terms of chemo-mechanical performance, but also enables cross-linked polymers to be reprocessed and recycled in a solid state. In the past decade, different vitrimers have been developed based on different exchangeable bonds, e.g., ester bond, disulfide and hindered urea bond [5,6,7,8,9]. Among them, epoxy-based transesterification is the most studied vitrimer chemistry due to its easy mass production [10,11,12,13]. Epoxy vitrimer is readily developed by adding an appropriate catalyst (such as zinc acetylacetonate (Zn(acac)_2_) [11], and triazobicyclodecene (TBD) [14], etc.) into classical formulations of epoxy, which have been widely used in the fields of aerospace, electrical engineering, and coatings [15]. However, one of the main limitations of the epoxy vitrimer is that the catalysts are toxic and corrosive, which may cause corrosion damage to materials [16]. To date, vitrimers-based polymer blends have not yet been studied systemically.

Epoxy vitrimer is intrinsically composed of ester bonds and hydroxyl groups, rendering bond exchange reactions (BERs) attainable, which makes it possible for them to be welded from a bulk state or ground powder state. Yu et al. [17,18,19] used a constant press force of kPa to weld and recycle soft epoxy vitrimer, and the recycled material restored the mechanical properties to the level of the fresh material. For hard epoxy vitrimer, the welding pressure increased to MPa [20]. Luzuriaga et al. ground the epoxy vitrimer composites into powder and obtained the second-generation composites under 20 MPa pressure [1]. Chabert et al. further proved the repeated welding ability of epoxy vitrimer composites in comparison with traditional thermosetting epoxy composites [21]. Compared with soft materials, chain diffusion in hard vitrimer poses significant difficulties and challenges due to the movement resistance of the densely cross-linked network. A large press force should be applied to make sure the welding surfaces are in full contact for welding. However, an excessive press force might wreak damage on the material due to its ductile nature.

Additionally, attempts have been made to lower the press force for welding epoxy vitrimer, where BERs were triggered between hydroxyl groups in alcohol solvent (e.g., EG, 2E1H) and esters in the epoxy network [22,23,24]. This type of BER containing a solvent is called a solvent-assisted BER and provides an efficient and mild way to recycle the polymer matrix, as well as the carbon fibers, with almost 100% recovered mechanical properties in a carbon fiber composite [23]. Based on this, Shi et al. [25] proposed a solvent-assisted welding strategy for the first time to realize welding under tiny press force, where, before welding, the surface of the soft epoxy vitrimer is depolymerized by an alcohol solvent. The essential degradation step prolonged the welding time; nevertheless, it provided an easy and effective bonding strategy to assemble a composite under smaller pressure or without pressure. To meet the increasing needs of industrial applications, hard materials with high cross-linking density are in great demand. To the best of our knowledge, there are, up to date, no publications on the welding of high-performance epoxy vitrimer.

In this paper, solvent-assisted BERs, which have been used for recycling rather than welding, are used to weld hard epoxy vitrimer. Ethylene glycol (EG), which offers hydroxyl groups, reacts with ester bonds of epoxy vitrimer reversibly. The vitrimer is firstly depolymerized and then forms a gel layer, in which the depolymerized polymer can diffuse across the welding interface easily. This will highly reduce the welding pressure. On the other hand, the depolymerized polymer can be repolymerized in the welding region, resulting in strong covalent bonds at the welding interface. This paper is organized as follows. In Section 1, we introduce the vitrimer and summarize the welding. In Section 2, the mechanism of surface depolymerizing and welding is demonstrated. In Section 3, we describe the welding procedures and characterizations. In Section 4, we present the results of the welding behavior of epoxy vitrimer. The focus of the study is to examine the suitable depolymerizing and welding conditions to achieve high welding strength alongside reducing the welding pressure. The concluding remarks are presented in Section 5.

## 2. Depolymerizing and Welding Mechanism

**Figure 1:**
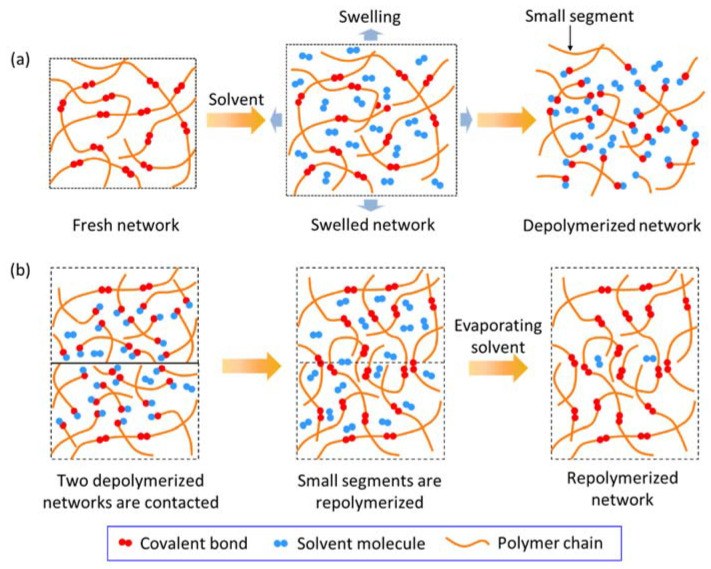
Schematic representation of depolymerizing and welding for vitrimer. (**a**) Original network is depolymerized into small segments. (**b**) Two depolymerized networks are repolymerized for welding.

The polymer network of vitrimer can be depolymerized into small segments, and further be repolymerized thanks to the reversibility of solvent-assisted BERs [22,23,24]. Accordingly, the depolymerizing and welding of vitrimer are shown in Figure 1. Vitrimer is firstly immersed into an excess amount of solvent, where the solvent molecules effectively diffuse into the polymer and swell the fresh network. Then, the swelled network is gradually depolymerized as the depolymerized network, where a long polymer chain is broken into some small segments, as shown in Figure 1a. The depolymerized network is taken out of the solvent. As shown in Figure 1b, as two pieces of the depolymerized network touch each other, the small segments diffuse across the welding interface and repolymerize. Meanwhile, the re-generated solvent molecules escape from the repolymerized network and gradually evaporate. Compared with long polymer chains in the fresh network, the small segments in the depolymerized network are prone to diffusion [25]. Moreover, the depolymerized network is in a viscous gel state [26], guaranteeing good contact. Therefore, the facile surface depolymerization generally benefits chain diffusion, promoting the welding of vitrimer under small press force.

## 3. Experimental

### 3.1. Materials

Diglycidyl ether of bisphenol A (DGEBA, MW: 340.4 g/mol, Sigma Aldrich, St. Louis, MO, USA), glutaric anhydride (GA, MW: 114.10 g/mol, Macklin, Shanghai, China), zinc acetylacetonate hydrate (Zn(ac)_2_, Sigma Aldrich, St. Louis, MO, USA), and ethylene glycol (EG, AR grade, Macklin, Shanghai, China).

### 3.2. Preparation of Hard Epoxy Vitrimer

Hard epoxy vitrimer was prepared with the following synthesis procedures: DGEBA and GA in solid states were heated at 130 °C until melted. Then, 5 mol % Zn(ac)_2_ to the epoxy groups was added into melted DGEBA and stirred powerfully at 130 °C for 10 min. Then, the melted GA was poured into the mixture with a stoichiometric ratio of epoxy groups and acyl groups of 1:1. The above mixture was poured into home-made molds to cure at 140 °C for 12 h after undergoing vacuum degassing at 100 °C for 15 min. The mechanical and thermal properties of the epoxy vitrimer are presented in Appendix A.

### 3.3. Dissolving Experiment

An appropriate depolymerization condition for welding was roughly obtained by a dissolving experiment for epoxy vitrimer. In our previous work [16], the dissolving temperature for epoxy vitrimer was located at 140–200 °C, which was around the boiling point of EG (197.3 °C) and far less than the pyrolysis temperature of the epoxy vitrimer (Table A1). Herein the dissolving temperature was 180 °C. The mass ratio of epoxy vitrimer to EG was 1:25. A cubic epoxy vitrimer sample with the length of 10 mm was immersed into EG solvent, then placed in an oven and heated under 180 °C. At different time intervals, the sample was taken out and weighed immediately to record the mass of the residual vitrimer.

### 3.4. Welding Experiments

**Figure 2:**
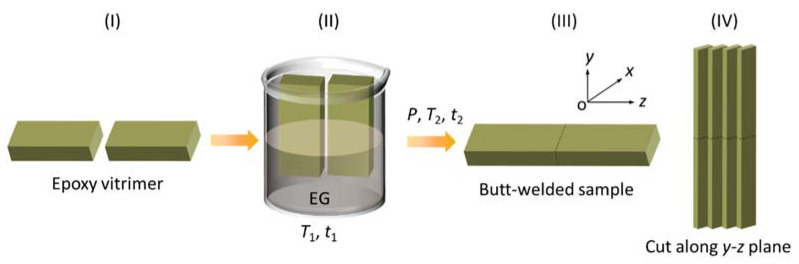
Schematic of the solvent-assisted welding process of epoxy vitrimer: (**I**) original epoxy vitrimer, (**II**) immersing into EG, (**III**) butt-welded vitrimer, and (**IV**) cut specimens for stretching.

**Figure 3:**
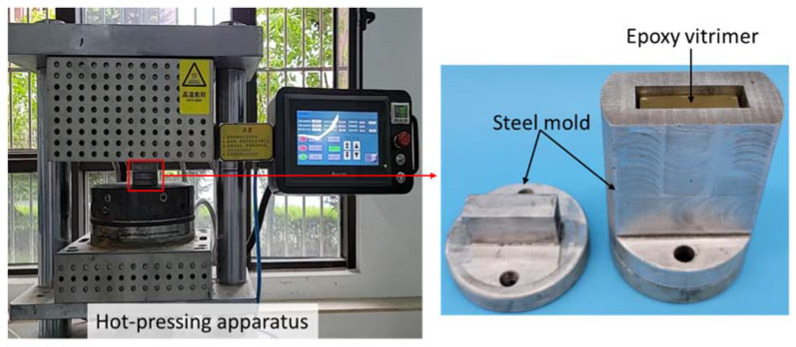
Experimental setup for welding.

The solvent-assisted welding process is shown in Figure 2. Two separated vitrimer samples (length 25 mm, width 15 mm, and thickness 8 mm) (Figure 2I) were put into EG solvent at a depolymerizing temperature of *T*_1_ for a depolymerizing time of *t*_1_ in a vacuum oven (Figure 2II). Then, two depolymerized vitrimer were connected under a welding pressure of *P* at a welding temperature of *T*_2_ for a welding time of *t*_2_, where the area of the butt joint was 15 mm × 8 mm (Figure 2III). All of the depolymerizing as well as welding conditions are summarized in Group 1–4 in Table 1. The welded vitrimer with a butt-joint was cut along the *y*-*z* plane to prepare stretching specimens with a thickness of ~2 mm (Figure 2IV). During welding, the welding pressure and welding temperature were provided by a hot-pressing apparatus (Figure 3), in which the heating rate and cooling rate were 20 °C/min and 10 °C/min, respectively. Moreover, a steel mold was fabricated to prevent the vitrimer sample from buckling during the welding process.

The dimension of the vitrimer for hot-press welding was the same as those for solvent-assisted welding. The hot-press welding process was carried out as follows. The epoxy vitrimer samples were cleaned with ethyl alcohol and then dried at 100 °C for 10 min to evaporate the ethyl alcohol. Then, the vitrimer samples were contacted using the hot-pressing apparatus and the steel mold (Figure 3). The welding pressure, welding temperature, and welding time are shown in Group 5 in Table 1.

### 3.5. Characterization

The butt-welded vitrimer was tested using a universal material testing machine (MTS Criterion C45.105, Eden Prairie, MN, USA) with a tensile rate of 1 mm/min. The force and crosshead displacement were recorded. The strain was calculated as the displacement divided by the initial distance of the crosshead. The stress was calculated as the force divided by the welding area in the undeformed state. Thus, the welding strength of epoxy specimens was calculated by:

(1) $$\tau =\frac{F_{\max}}{S}$$

where *F*_max_ was the maximum load and, *S* was the welding area in the undeformed state. At least three specimens were tested for each case.

The surface morphologies of the welded region were investigated by a 3D microscope system (LY-WN-YH System, Liyang Co., Ltd., Chengdu, Sichuan, China).

## 4. Results and Discussions

### 4.1. The Influence of Depolymerizing Time

**Figure 4:**
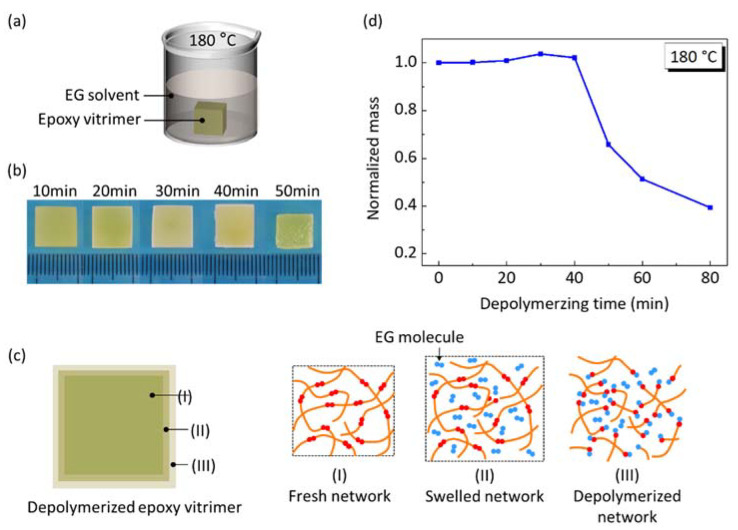
Depolymerization of epoxy vitrimer being immersed into EG at 180 °C. (**a**) Dissolving experiment of the vitrimer. (**b**) The appearance and size evolution of the depolymerized vitrimer. (**c**) Schematic representation of the depolymerized vitrimer. (**d**) Normalized mass of depolymerized vitrimer as a function of depolymerizing time.

The depolymerizing time for welding was determined by the dissolving experiment of cubic epoxy vitrimer into EG solvent at 180 °C, as shown in Figure 4a. During dissolving, the EG solvent provided abundant hydroxyl groups to break the esters on the polymer network, leading to depolymerization of the vitrimer. The dissolving process involved three processes [16], e.g., diffusion of EG molecules into the network, the cleavage of the polymer chains due to solvent-assisted BERs between the hydroxyl groups of the EG solvent and esters of the network, and the diffusion of small segments into the EG solvent. Figure 4b shows that the cubic shape of the vitrimer remains unchanged during the dissolving process, indicating that the dissolution of the vitrimer is a typical surface erosion mode [22,26].

After partially dissolving, the depolymerized vitrimer was assumed to contain three parts: the fresh network, the swelled network, and the depolymerized network, as shown in Figure 4c. The swelled network and the depolymerized network are much smaller than the fresh network. In the swelled network, a small amount of EG molecules are absorbed but do not break the network. In the depolymerized network, the network is depolymerized into small segments due to solvent-assisted BERs. Therefore, the mass of the depolymerized vitrimer is the total mass containing absorbed EG molecules in the swelled network and depolymerized network. Here, the mass of the depolymerized vitrimer is normalized by the initial mass, as shown in Figure 4d. In the first 30 min, the mass of the depolymerized vitrimer increases slightly due to the diffusion of EG molecules in the swelled network; however, the depolymerized network is very small. When the dissolving time increases from 30 min to 40 min, the absorbed EG continues to increase but the depolymerized mass is still small. The mass of the depolymerized vitrimer decreases sharply after dissolving for 40 min, in which there is an obvious material loss. In conclusion, the surface depolymerization has dual characteristics for welding. On the one hand, it contributes to breaking the fresh network, which will promote the followed welding process. On the other hand, it gives rise to vitrimer loss, which will be detrimental to the vitrimer integrity.

**Figure 5:**
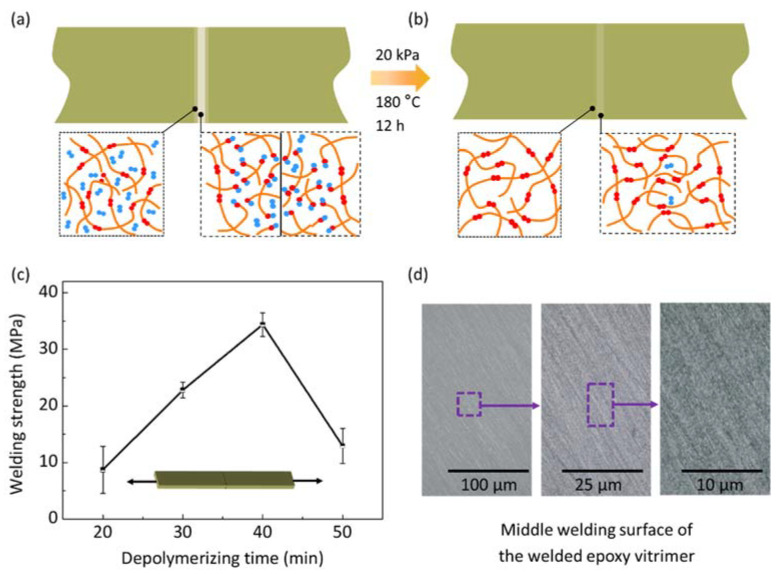
Effect of depolymerizing time on the welding performance of epoxy vitrimers. The vitrimer are welded under 20 kPa at 180 °C for 12 h. Schematic representation of the depolymerized vitrimers (**a**) before welding and (**b**) after welding. (**c**) The welding strength of the butt-welded vitrimer versus depolymerizing time. Each data point represents the mean and stands deviation of three measurements. (**d**) The microscopical images of welded region with depolymerizing time of 40 min.

To quantitatively provide criteria for optimizing the depolymerizing time, we welded the depolymerized vitrimer and compared their welding strength. At the beginning of the welding, two pieces of the depolymerized vitrimer specimens are connected to each other (Figure 5a). The depolymerized network is diffused across the interface. After being welded, the absorbed EG molecules in the swelled network are evaporated, as shown in Figure 5b. Meanwhile, the generated solvent molecules evaporate from the interface. Thus, the depolymerizing conditions affect the degree of depolymerization, further affecting the welding strength. For a given depolymerizing temperature, the welding strength is only related to the depolymerizing time.

After being depolymerized for 20 min, 30 min, 40 min, and 50 min, respectively, the depolymerized vitrimer were welded at 180 °C under 20 kPa for 12 h (Group 1 in Table 1). The tensile behavior of the butt-welded specimens is shown in Figure 5c. The greatest welding strength is about 35 MPa for a depolymerizing time of 40 min (54% of the fresh vitrimer), while the smallest welding strength is about 8 MPa for a depolymerizing time of only 20 min. The welding strength decreases to 12 MPa as the depolymerizing time increases to 50 min, which is lower than that for a depolymerizing time of 30 min and 40 min. The trend that the welding strength increases at first and then declines with depolymerizing time can be explained. On the one hand, the content of EG absorbed in the polymer network is very limited within 20 min, which is not beneficial to repolymerization for the subsequent welding process. On the other hand, the surface of the vitrimer is over-depolymerized for 50 min, making repolymerization difficult due to the imperfect surface contact. The vitrimer under a depolymerizing time of 40 min possesses the largest swelling content of EG, which improves depolymerization, while no severe fracturing of the vitrimer occurs.

Figure 5d shows the microscopic images of the welded region with a depolymerizing time of 40 min and a welding time of 12 h. It was found that there is no obvious welding interface from the middle surface view, which reflects efficient welding with zero-interface. However, the failure of the welded specimen substantially originates from the welding interface because incomplete repolymerization with insufficient welding conditions was provided.

### 4.2. The Influences of Welding Conditions

**Figure 6:**
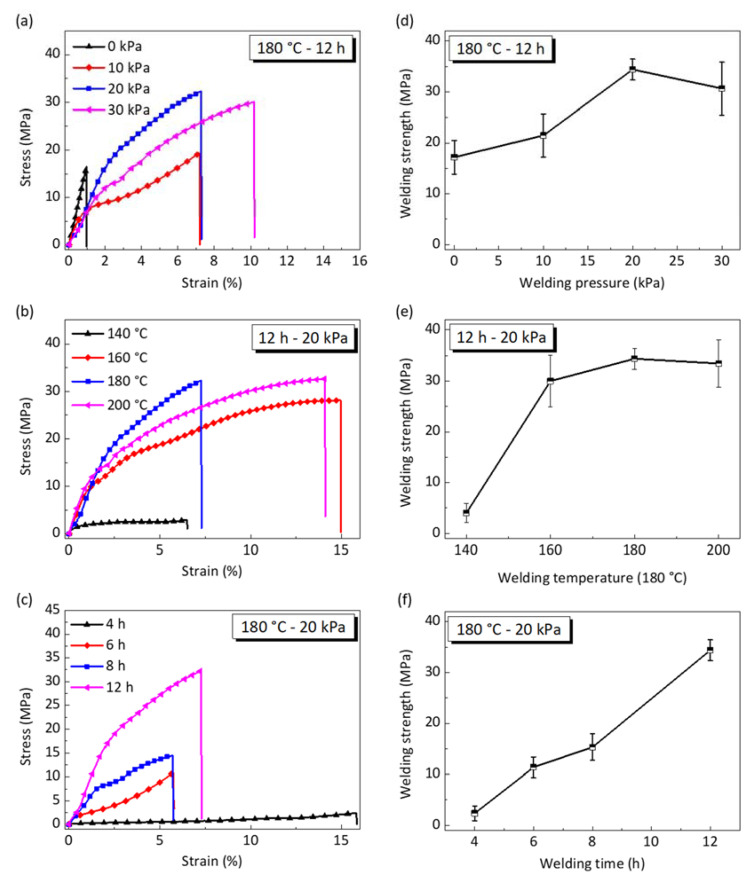
Effect of welding conditions on the mechanical behavior of butt-welded epoxy vitrimer. All of the epoxy vitrimers are depolymerized at 180 °C for 40 min before welding. Stress–strain curves under different (**a**) welding pressure, (**b**) welding temperature, and (**c**) welding time. The welding strength varies with (**e**) welding pressure, (**e**) welding temperature, and (**f**) welding time. Each data point represents the mean and stands deviation of three measurements.

The depolymerized network will be re-polymerized due to the solvent-assisted BERs, which can be reversed using the depolymerizing process [23,24,25]. According to our previous work for recycling epoxy, visible hydroxyl groups unavoidably exist in the repolymerized network and the residual EG molecules substantially reduce the degree of the repolymerized network [16]. Thus, the welding quality highly depends on the welding conditions, e.g., welding pressure, welding temperature and welding time. Herein we study how the above welding conditions affect the welding strength, and the corresponding results are shown in Figure 6. Before welding, the vitrimer samples are depolymerized at 180 °C for 40 min.

Figure 6a, d show the influence of welding pressure on stress–strain curves and the welding strength, respectively. The welding temperature is 180 °C and the welding time is 12 h (Group 2 in Table 1). The welding strength is 17.2 MPa for the case with a lack of welding pressure. As the welding pressure increases to 20 kPa, the welding strength increases to 34 MPa, indicating that a higher press force increases the contact area and further enhances the welding strength. However, the welding strength decreases slightly as the welding pressure increases to 30 MPa. This is possibly because excess press force squeezes out the depolymerized network and further prevents depolymerized chain diffusion at the welding interface. Therefore, increasing the press force does not significantly promote the welding efficiency, which is completely different from the reported results, indicating that press force accelerates welding without surface depolymerization [19,20].

Figure 6b,e show the influence of welding temperature on stress–strain curves and the welding strength, respectively. The welding pressure is 20 kPa and the welding time is 12 h (Group 3 in Table 1). The welding strength is only 4 MPa when the welding temperature is 140 °C, and reaches 35 MPa as the temperature approaches 180 °C. This is because higher temperature promotes solvent-assisted BERs and, likewise, EG evaporation simultaneously. The degree of repolymerized network enlarges while the residual EG lessens, resulting in improved welding strength. As the welding temperature increases to 200 °C, the welding strength is slightly lower than that at 180 °C. This is probably ascribed to the fact that the faster formation of the repolymerization network on the welding surface prevents the evaporation of enclosed EG molecules and further lowers the welding strength. Moreover, excessive welding temperature may lead to surface degradation due to oxidation.

Figure 6c, f show that the stress–strain curves and welding strength vary with a welding time of 4 h, 6 h, 8 h, and 12 h (Group 4 in Table 1). The welding pressure and welding temperature are 20 kPa and 180 °C, respectively. It is seen that the welding strength gradually increases as the welding time increases. Sufficient welding time ensures the full completion of network repolymerization at the welding interface, which enables strong welding. Moreover, extending the welding time facilitates EG evaporation in the repolymerized network at the welding interface, which finally boosts the welding strength. Thus, we believe that a higher welding strength could be achieved if we further increase the welding time.

### 4.3. Comparison to Welding with no Surface Depolymerization

**Figure 7:**
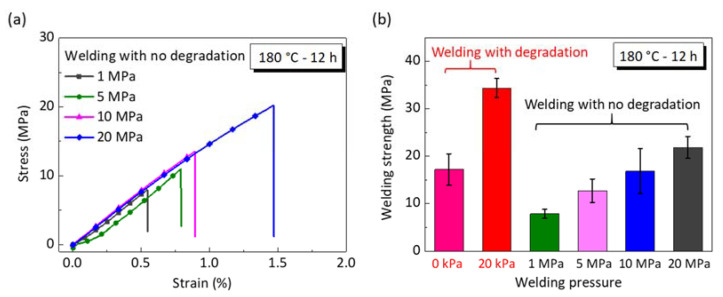
Comparison of the welding performance of epoxy vitrimer using hot-press welding and solvent-assisted welding. (**a**) Stress–strain curves of hot-press welding. (**b**) Welding strength of solvent-assisted welding and hot-press welding. The welding time and temperature are 12 h and 180 °C, respectively. Each data point represents the mean and stands deviation of three measurements.

For comparison, the hard epoxy vitrimer are welded using hot-press welding without surface depolymerization, where the welding pressure is from 1 MPa to 20 MPa. Figure 7a shows the stress–strain curves of welded vitrimer as they are welded at 180 °C for 12 h (Group 5 in Table 1). All the curves are linear and have the same slope. Compared with Figure 6a,c, one can see that the break strain using hot-press welding is much smaller than that using solvent-assisted welding. In addition, the comparison of welding strength with and without surface depolymerization is shown in Figure 7b. Without surface depolymerization, welding under a large pressure of 10 MPa reaches to a welding strength of 16.8 MPa, close to the strength level of welding under zero pressure as the vitrimer is depolymerized. The maximum welding strength of 35 MPa is attained after surface depolymerization, 1.55-times higher than the welding at an extreme press force of 20 MPa without surface depolymerization, whereas the welding pressure of the former is 1/1000 of the latter. Therefore, we can conclude that the use of surface depolymerization encourages the fluidity of the polymer chains to diffuse readily, which further strikingly reduces the required press force for welding.

Epoxy vitrimer can be intrinsically welded due to the self-contained dynamic covalent bonds [27]. In comparison with soft materials, the required welding pressure for vitrimers with high cross-linking densities should be larger as the polymers are too inactive to deform [17]. This work introduces a facile surface depolymerization strategy to reduce the welding pressure for welding high-performance epoxy vitrimer. The previous welding method has the following two advantages. On the one hand, the depolymerized materials on the surface of the vitrimer form a gel layer with a thickness of ~0.1 mm, which is prone to diffusing across the welding interface [25]. As a consequence, the welding pressure, which is used to make sure the welding surfaces come into contact, decreases from tens of megapascals to tens of kilopascals, as shown in Figure 7. On the other hand, large numbers of hydroxyl groups are introduced, which accelerate the bond exchange reaction and form covalent bonds at the welding interface, thus promoting strong welding (Figure 1). In view of the above-mentioned advantages, this surface degradation strategy has the potential for welding vitrimer composites to meet the increasing needs of industrial applications [27,28,29]. However, there are still some problems in the surface degradation for welding, such as the need for high welding temperatures, which are determined by the BERs and the boiling point of the EG. Therefore, it is worth further exploring solvents with low boiling points as well as more efficient BER chemistry for relatively low temperatures.

## 5. Conclusions

We have realized strong welding for hard epoxy vitrimer under small press force thanks to facile surface depolymerization. The vitrimer specimens are firstly dissolved into an excess amount of EG solvents for surface depolymerization and then welded as the depolymerized vitrimer specimen pieces touch each other. We first conducted dissolving experiments and welding experiments to determine the depolymerizing time of epoxy vitrimer, which is 40 min under 180 °C. Then, we investigated the influence of welding conditions on the welding strength of epoxy vitrimer. During the welding process, an appropriate welding temperature and long welding time are favored in attaining remarkable welding strength. Furthermore, increasing the welding pressure does not significantly promote the welding efficiency, which is completely different from the reported results of hot-press welding. Under the same conditions (180 °C, 12 h), the welding strength using surface depolymerization is 35 MPa under 20 kPa, while that using hot-pressing welding is only 21.9 MPa. That is, the welding strength using surface depolymerization is 1.55-times higher, but the magnitude of press force is 1/1000. This work provides an example of using surface depolymerization to develop welding methods for vitrimer composites. Nevertheless, these are comparatively high welding temperatures determined by the boiling point of EG. Therefore, it is worth further exploring solvents with low boiling points.

## Figures and Tables

**Table 1:** Depolymerizing and welding conditions for epoxy vitrimer.

Group	Depolymerizing Time *t*_1_ (min)	Depolymerizing Temperature *T*_1_ (°C)	Welding Pressure *P* (kPa)	Welding Temperature *T*_2_ (°C)	Welding Time *t*_2_ (h)
Group 1	20, 30, 40, 50	180	20	180	12
Group 2	40	180	0, 10, 20, 30	180	12
Group 3	40	180	20	140, 160, 180, 200	12
Group 4	40	180	20	180	4, 6, 8, 10, 12
Group 5	/	/	1000, 5000, 10,000, 20,000	180	12

**Figure A1:**
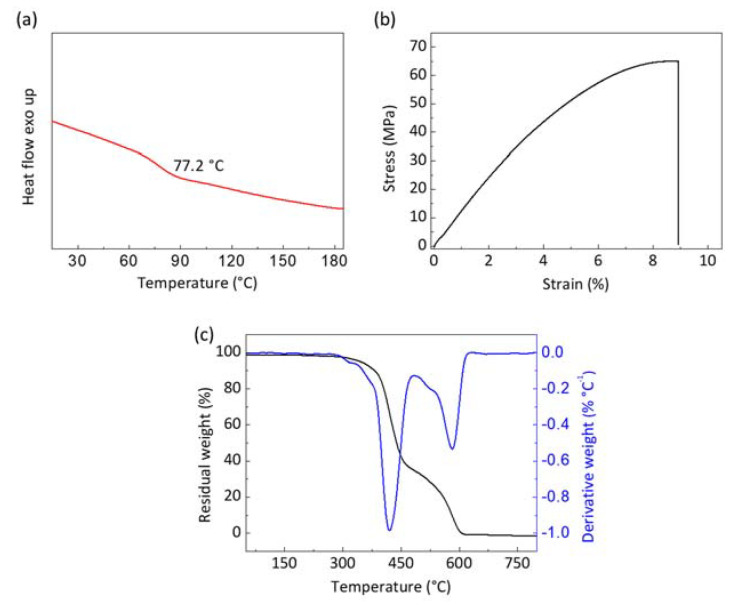
The mechanical and thermal properties of epoxy vitrimer. (**a**) DSC curve, (**b**) Stress–strain curve, (**c**) TGA and DTG curves.

## Data Availability

The data presented in this study are available on reasonable request from the corresponding author.

## Appendix A. The Mechanical and Thermal Properties of Hard Epoxy Vitrimer

DSC measurements were conducted using a DSC1 differential scanning calorimeter (Mettler Toledo, Switzerland). The sample was heated from 0 °C to 250 °C at a rate of 10 °C/min. The DSC curve of epoxy vitrimer shows an obvious glass transition of 77.2 °C in the second heating curve, as shown in Figure A1a. The glass transition temperature is also list in Table A1.

Tensile tests of epoxy vitrimer were carried out using a universal material testing machine (MTS Criterion C45.105, Eden Prairie, MN, USA). The epoxy vitrimer samples were cut into a dog-bone shape and tested according to ASTM standard 638-14. The tensile stress–strain curve of epoxy vitrimer is shown in Figure A1b. The basic mechanical parameters are listed in Table A1.

Thermogravimetric analysis (TGA) was performed using a TGA instrument (Netzsch STA449F5, Germany). The experiments were performed within an air atmosphere at a ramp rate of 10 °C/min from 30 °C to 800 °C, where the gas flow rate was 50 mL/min. TGA profiles (Figure A1c) show that the decomposition temperatures exhibits two stages: the first one ranges from 330 °C to 482 °C due to pyrolysis and carbonization; while the second stage varies from 482 °C to 607 °C due to the decomposition of the carbonized products. All the thermal and mechanical properties are summarized in Table A1.

**Table A1:** Thermal and mechanical properties of the cured epoxy vitrimer.

*T*_g_/°C	Tensile Strength/MPa	Elastic Modulus/MPa	Elongation at Break/%	*T*_5%_/°C	*T*_10%_/°C	*T*_max1_/°C	*T*_max2_/°C
77.2	63.0 ± 3.4	1192.9 ± 34.5	8.6 ± 2.4	346	382	420	582
